# Self-Supervised Machine Learning to Characterize Step Counts from Wrist-Worn Accelerometers in the UK Biobank

**DOI:** 10.1249/MSS.0000000000003478

**Published:** 2024-05-15

**Authors:** SCOTT R. SMALL, SHING CHAN, ROSEMARY WALMSLEY, LENNART VON FRITSCH, AIDAN ACQUAH, GERT MERTES, BENJAMIN G. FEAKINS, ANDREW CREAGH, ADAM STRANGE, CHARLES E. MATTHEWS, DAVID A. CLIFTON, ANDREW J. PRICE, SARA KHALID, DERRICK BENNETT, AIDEN DOHERTY

**Affiliations:** 1 Nuffield Department of Population Health, University of Oxford, Oxford, UNITED KINGDOM; 2 Big Data Institute, Li Ka Shing Centre for Health Information and Discovery, University of Oxford, Oxford, UNITED KINGDOM; 3 Nuffield Department of Orthopaedics, Rheumatology and Musculoskeletal Sciences, University of Oxford, Oxford, UNITED KINGDOM; 4 Institute of Biomedical Engineering, Department of Engineering Science, University of Oxford, Oxford, UNITED KINGDOM; 5 SwissRe Institute, UNITED KINGDOM; 6 Division of Cancer Epidemiology and Genetics, National Cancer Institute, Rockville, MD

**Keywords:** MACHINE LEARNING, STEP DETECTION, CADENCE, ACTIVITY, MORTALITY, VALIDATION

## Abstract

**Purpose:**

Step count is an intuitive measure of physical activity frequently quantified in health-related studies; however, accurate step counting is difficult in the free-living environment, with error routinely above 20% in wrist-worn devices against camera-annotated ground truth. This study aimed to describe the development and validation of step count derived from a wrist-worn accelerometer and assess its association with cardiovascular and all-cause mortality in a large prospective cohort.

**Methods:**

We developed and externally validated a self-supervised machine learning step detection model, trained on an open-source and step-annotated free-living dataset. Thirty-nine individuals will free-living ground-truth annotated step counts were used for model development. An open-source dataset with 30 individuals was used for external validation. Epidemiological analysis was performed using 75,263 UK Biobank participants without prevalent cardiovascular disease (CVD) or cancer. Cox regression was used to test the association of daily step count with fatal CVD and all-cause mortality after adjustment for potential confounders.

**Results:**

The algorithm substantially outperformed reference models (free-living mean absolute percent error of 12.5% vs 65%–231%). Our data indicate an inverse dose–response association, where taking 6430–8277 daily steps was associated with 37% (25%–48%) and 28% (20%–35%) lower risk of fatal CVD and all-cause mortality up to 7 yr later, compared with those taking fewer steps each day.

**Conclusions:**

We have developed an open and transparent method that markedly improves the measurement of steps in large-scale wrist-worn accelerometer datasets. The application of this method demonstrated expected associations with CVD and all-cause mortality, indicating excellent face validity. This reinforces public health messaging for increasing physical activity and can help lay the groundwork for the inclusion of target step counts in future public health guidelines.

Physical activity has been associated with a lower risk of a wide range of noncommunicable diseases and is a key feature of public health guidelines for cardiovascular health (1–3). Although researchers most commonly report device-measured activity in terms of overall acceleration or time-use behaviors derived from intensity thresholds (4), the reporting of steps is a more intuitive measure of physical activity intrinsically linked to the key biomechanical feature of human gait (5). However, current methods to measure steps from wrist-worn monitors during free-living activity are inaccurate when compared with camera annotated ground truth (6).

Most activity tracking devices rely on proprietary step counting methods without transparent evaluation (7), and many popular open-source step counting algorithms were not developed in accordance with, or lack validation against, direct observation ground-truth step counts in a free-living environment (8–10). Current standards require commercial activity trackers to estimate step counts with an error of less than 10% in laboratory-controlled treadmill testing (11). Subsequently, many devices and algorithms perform well during scripted, moderately paced walking in controlled conditions (12,13). However, step counting performance substantially deteriorates in the real-world environment, wherein mean absolute percent error (MAPE) is regularly well above 20% in both commercial and research-grade activity monitors during free living (6). No standards of performance are placed on research-grade devices, wherein researchers can freely apply manufacturer-provided, open-source, or bespoke step-detection algorithms to the raw acceleration data collected by these devices, which can lead to substantial under or overreporting of steps depending on the algorithm used (6). As a consequence, uncertainty exists around the strength and shape of the association of daily step count with all-cause mortality and cardiovascular mortality (14,15), where recent studies have not used transparent or robustly validated free-living step counting algorithms for wrist-based activity measurement.

Here, we develop a hybrid self-supervised machine learning method to accurately measure steps in free-living environments. We validate this method in cross-validation and external datasets and demonstrate superior performance of the new methods in comparison with other open-source step detection algorithms. We then establish face validity of this method in the large scale prospective UK Biobank cohort study by associating step counts with fatal cardiovascular disease (CVD) and all-cause mortality. Finally, we develop an open-source software package for free use by the research community to accurately quantify steps from raw, free-living wrist-worn accelerometer data.

## METHODS

### Development of the free-living, ground-truth annotated OxWalk dataset

To develop the OxWalk (16) dataset, participants contributed activity data during unscripted, free living. Ethical approval for OxWalk participant recruitment was obtained from the Central University Research Ethics Committee of the University of Oxford (Ref: R63137/RE001). Written informed consent was obtained from adult volunteers (aged 18 yr and above) with no lower limb injury within the previous 6 months and who were able to walk without an assistive device. Participants wore four triaxial accelerometers (AX3; Axivity, Newcastle, UK), two placed side-by-side on the dominant wrist and two clipped to the dominant-side hip at the midsagittal plane. Accelerometers were synchronized using the Open Movement GUI software (v.1.0.0.42), with one recording at 100 Hz and the other at 25 Hz at each body location. Final accelerometer data were resampled to the nominal sampling rate and calibrated to local gravity using the Open Movement software package. Foot-facing video was captured using an action camera (Action Camera CT9500; Crosstour, Shenzhen, China) mounted at the participant’s beltline (Supplemental Fig. 1, A foot-facing action camera clipped at the participant’s waistline recording video for step count annotation, http://links.lww.com/MSS/D29). Participants were instructed to wear the camera for 1 h and could remove the camera any time they felt uncomfortable or required additional privacy (17). To create a clear, easily distinguishable data point for video and accelerometer synchronization in this study, participants were asked to strike their accelerometers together with four forceful blows within camera view at the start of data collection (18).

Ground-truth annotation of steps was conducted within video annotation software (Elan 6.0; The Language Archive, Nijmegen, the Netherlands) by two independent annotators (S. S. and L. v. F.) blinded to each other’s results. Similar to Bassett et al. (5), we identified the act of lifting a foot and placing it in a new location as a central tenant of step identification. This definition was used as the framework for step annotation in the OxWalk dataset, with an annotated step being a repositioned foot linked to a change in gross body position along the floor. Annotated steps did not include foot shuffling, changing of foot alignment via pivoting, or shifting of weight from one foot to the other.

### Model development and evaluation

**FIGURE 1:**
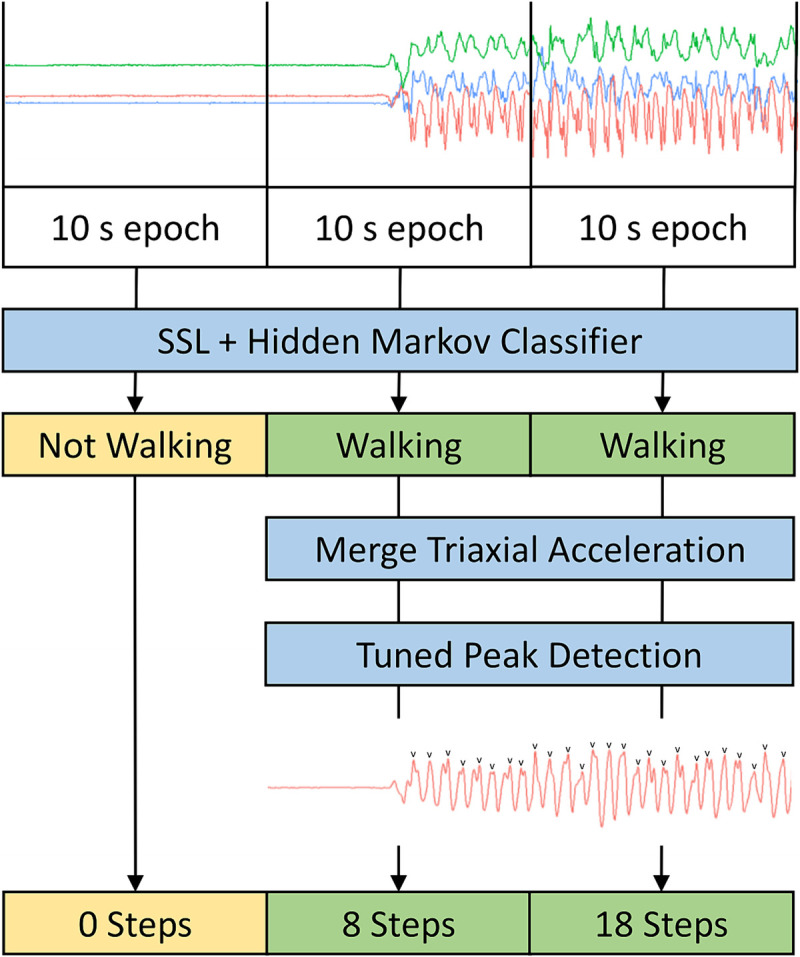
Schematic of the process for generating step count from 30 s of raw triaxial accelerometer data using a hybrid SSL and peak detection step counting model.

To develop the proposed step count model, a hybrid machine learning and peak detection algorithm was created wherein an activity classification model was first used to detect periods of walking and non-walking, followed by step counting only on predicted walking data epochs (Fig. 1). Activity classification was performed using a self-supervised deep learning model developed by Yuan et al. (19) incorporating an 18-layer ResNet-V2 (20) pretrained using self-supervised tasks on the UK Biobank accelerometer dataset. This pretraining step has previously demonstrated consistent performance improvement for downstream activity recognition tasks against Random Forest activity classification (19). The pretrained self-supervised learning (SSL) model was then trained for supervised gait classification using the OxWalk dataset, wherein training data consisted of 10-s epochs of accelerometer data with ground-truth walk or non-walk labels. In the OxWalk dataset, walking was defined as at least four steps within the 10-s epoch. Ten-fold cross-validation was used to train and validate the walking activity classifier and evaluate end-to-end performance of the step detection pipeline. The participant dataset was divided into 10 equal random folds where one fold was left out for testing and the remaining folds underwent a randomized 80%–20% split for training and validation, respectively. Folds were stratified by class label, and data were grouped by participant. The SSL model was trained on the remaining set with an early-stopping mechanism on the validation set when the loss stopped decreasing for five consecutive training epochs. The weights before early stopping were used to perform activity prediction on the test and validation set. An additional data-augmentation step was performed during training, whereby each triaxial training sample was randomly transformed with a rotation along a random axis and the axes were switched in a random order to make the model rotation invariant. The model was trained using PyTorch 1.12.1 and Adam optimization (21) with a learning rate of 0.0001. Weighted cross entropy loss was used, with the class weights set in such a way that the balance of walking and non-walking segments was 10% to 90%, respectively, bringing the class balance in line with 24-h direct observation during free living in a previously collected dataset (22). Finally, predictions on the validation set and corresponding ground-truth labels were used to train a Hidden Markov Model smoother, which was then applied to the predictions in the test set.

Step counting was performed through peak detection on classified walking time windows using the “find_peaks” method from the SciPy Python package (23). Euclidean norm of triaxial acceleration, minus 1*g* to remove the effect of gravity, was clipped between ±2*g* and filtered using a low-pass fourth-order Butterworth filter at 5 Hz before use as the input signal for peak detection. The “find_peaks” method detects local peaks using predefined heuristics including the minimum peak height (prominence), maximum peak width (width), and minimum time between peaks (distance). These heuristics served as detection hyperparameters for which optimal values would minimize the mean absolute error for step count in the validation set. Detection parameters were iterated across a preselected range of values (prominence: 0.1 to 1*g*; distance: 0.2 to 2 s; width: 10 ms to 1 s).

Model performance metrics were calculated on participants within each test set; mean precision, recall, F1, Cohen’s kappa, and accuracy were used to evaluate walking classification, whereas MAPE and mean bias and Spearman’s rank correlation coefficient were calculated against ground-truth step annotations. Following internal model validation, the final activity prediction model was retrained on the entire OxWalk dataset with an 80%–20% training-validation split before external deployment.

### External model validation

External model performance was assessed by applying the step detection algorithm to wrist-worn accelerometer data to an open-source, step-annotated dataset from Clemson University (24). Within this external dataset, 30 participants contributed a mean of 37 min of activity, split between three distinct sessions of regular walking (two laps around a predefined path), semiregular walking (locating objects throughout a building), and irregular walking (collecting and assembling building blocks distributed around a room). Participants were video recorded throughout scripted activities, allowing timestamp-annotated steps while wearing Shimmer3 inertial measurement units (Shimmer, Dublin, Ireland) recording at 15 Hz. Researchers annotated steps as well as “shifts,” foot movement not necessarily tied to a change in body position, although these annotated shifts were not included in the current analysis (24). Prediction error was quantified by calculating MAPE and mean percent under/overcounting bias for each gait subtype and overall, at the participant level, across all gait subtypes. Bland–Altman plots were created for comparison between cumulative ground truth and predicted step counts for each participant.

### Open-source step count algorithm assessment

In addition to assessment of the novel hybrid self-supervised algorithm, two additional step counting approaches were evaluated in this study using both the OxWalk and Clemson datasets: 1) a recently published acceleration-threshold algorithm by Ducharme et al. (8), and 2) the Verisense algorithm, a popular open-source peak detection algorithm developed from the Clemson dataset (25) and previously applied to UK Biobank accelerometer data using integration with the GGIR package (14,26). Further details for these algorithms are presented in Supplemental Note 1 (Comparative step detection models, http://links.lww.com/MSS/D29), whereas details of all datasets used are presented in Supplemental Table 1 (Summary of datasets, http://links.lww.com/MSS/D29).

### Model implementation into the UK Biobank

The UK Biobank is a prospectively recruited observational cohort of over 500,000 participants aged 40–69 yr at the time of recruitment, from 2006 to 2010 (27). From 2013 to 2015, participants were invited to wear an Axivity AX3 accelerometer on their dominant wrist, recording at 100 Hz, for a 7-d, 24-h·d^−1^ activity measurement window. In the current study, raw accelerometer data were processed from 103,614 available participants, after which data were excluded from participants with fewer than 72 h of wear, those lacking data across the entire diurnal cycle, with poor device calibration, or with unrealistic average acceleration (>100 m*g*) (4). The externally validated hybrid SSL step detection model was implemented into the OxWearables Stepcount package (v.2.1.5; University of Oxford, Oxford, UK, https://github.com/OxWearables/stepcount) and applied to raw accelerometer data from the UK Biobank. Overall daily step count was reported as the median number of steps taken across the 7-d measurement period. Missing step count data from nonwear were imputed by averaging step count from the corresponding time of day in all other valid days, similar to the imputation of vector magnitude acceleration during nonwear in the UK Biobank physical activity cohort (4). One-minute peak cadence was calculated, as previously described by Saint-Maurice et al. (28), as the mean of the single minute with the highest cadence value across each measured day.

### Epidemiological analysis for the association of step counts with all-cause and cardiovascular mortality

UK Biobank participants with prevalent CVD or cancer as a primary diagnosis, as identified by *International Classification of Diseases* codes I00–I99 and C00–C97 in their routine hospital data, were removed from analysis. Spearman’s rank correlation (*r*) was calculated between step count, peak cadence, overall acceleration, and UK Biobank–derived activity time use activity classification (22). Daily step count and 1-minute peak cadence were stratified across demographic and self-reported health variables as collected by the UK Biobank at the time of enrolment. Analysis of variance and Tukey honestly significant difference tests were conducted to compare step count based on self-reported health and usual walking pace.

Multivariable adjusted estimates of the effect of quintiles of step count on the relative hazards of cardiovascular mortality and all-cause mortality were derived using Cox proportional hazards regression using age as the underlying timescale (29,30). Date and cause of death were gathered from the UK Biobank linked death registry. Length of follow-up was calculated from censoring dates from the data sources or date of death. Further detail is provided in Supplemental Notes 2 and 3 (Statistical analysis, further detail, and Field and code usage, http://links.lww.com/MSS/D29). Step count detection was deployed on the UK Biobank using the University of Oxford Biomedical Research Computing cluster, whereas statistical analysis was completed using R (v.4.1.1) on the UK Biobank Research Analysis Platform.

## RESULTS

### Internal model validation and algorithm comparison in the OxWalk dataset

To create a free-living training and validation dataset, accelerometer and ground-truth camera data were collected from 39 participants (19 female, 20 male) with a mean age of 38.5 yr (range, 19.5–81.2 yr). Thirty-three participants were annotated by two annotators, resulting in a corresponding step count MAPE of 4.0% and interclass correlation coefficient of 1.0 between annotators.

**FIGURE 2:**
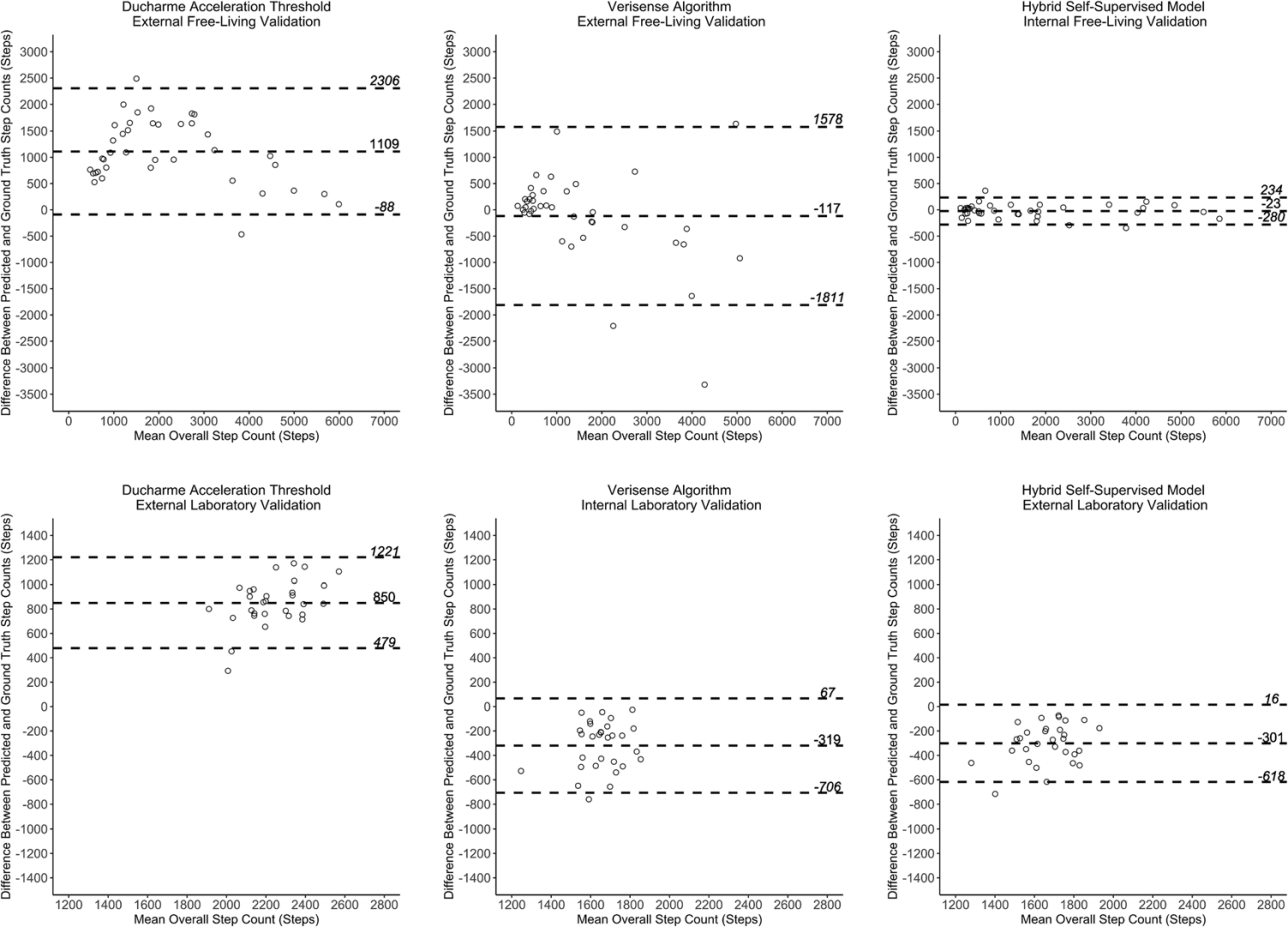
Bland–Altman plots with dotted 95% limits of agreement for the comparison of step counting models in the (top) OxWalk free-living dataset of 39 adults and (bottom) Clemson laboratory-based dataset of 30 young adults. Left: Ducharme baseline acceleration threshold model (8), centre: Verisense algorithm (25), and right: the novel hybrid SSL model. The Clemson dataset serves as an internal validation for the Verisense algorithm and an external validation for the hybrid SSL model. The OxWalk dataset serves as an external validation for the Verisense algorithm and an internal validation for the Hybrid SSL model. Both datasets serve for external validation of the Ducharme algorithm.

Internal validation of this SSL model identified bouts of walking with a Cohen’s kappa performance of 0.79 (Supplemental Table 2, Internal validation gait classification metrics for the SSL model, http://links.lww.com/MSS/D29). Overall cross-validation of step detection in the SSL model resulted in a 12.5% MAPE, 1.3% underestimation of steps, and correlation of *r* = 0.98 against ground truth in our free-living “OxWalk” dataset. For comparison, a previously published acceleration-threshold algorithm (8) resulted in a 69.1% overestimation of steps (231.3% MAPE, *r* = 0.91) across all participants. External validation of the Verisense algorithm (10,25), incorporated into recent UK Biobank papers (14,26), produced a 63.5% MAPE, 7.2% underestimation bias, and *r* = 0.85 against free-living ground-truth step counts (Supplemental Table 3, Step count results and performance metrics based in the OxWalk dataset, http://links.lww.com/MSS/D29). Bland–Altman plots for model comparisons against ground-truth step count are presented in Figure 2, demonstrating lower variability and tighter agreement with ground truth using our step detection algorithm in the free-living dataset.

### External model validation and algorithm comparison in the Clemson dataset

Bland–Altman plots for the performance of our SSL method and two reference algorithms in the overall Clemson dataset are also presented in Figure 2. This plot again demonstrates reduced variability and bias against ground truth using the hybrid self-supervised model compared with reference algorithms. In external validation, the threshold model by Ducharme et al. (8) performed well during sessions of regular gait, but poorly for irregular gait, culminating in an overall MAPE of 47.5% and a 46.9% overestimation of steps at the participant-level, across all gait subtypes. The Verisense algorithm, for which the Clemson dataset serves as an internal validation, demonstrated a 17.6% underestimation of steps and a 17.3% per-participant MAPE over all gait subtypes, including 16.3% MAPE during regular walking (Supplemental Table 4, Step count results and performance metrics based in the external Clemson Shimmer3 dataset across all participants and gait subtypes, http://links.lww.com/MSS/D29). External validation of our SSL hybrid step algorithm performed best in the Clemson dataset, producing a 16.5% MAPE and 16.6% underestimation across all gait subtypes, including 9.2% MAPE during regular walking. Because of superior performance in free-living and laboratory-based validation, the SSL step detection model was selected for analysis of UK Biobank data.

### Step counts in the UK Biobank physical activity cohort

**FIGURE 3:**
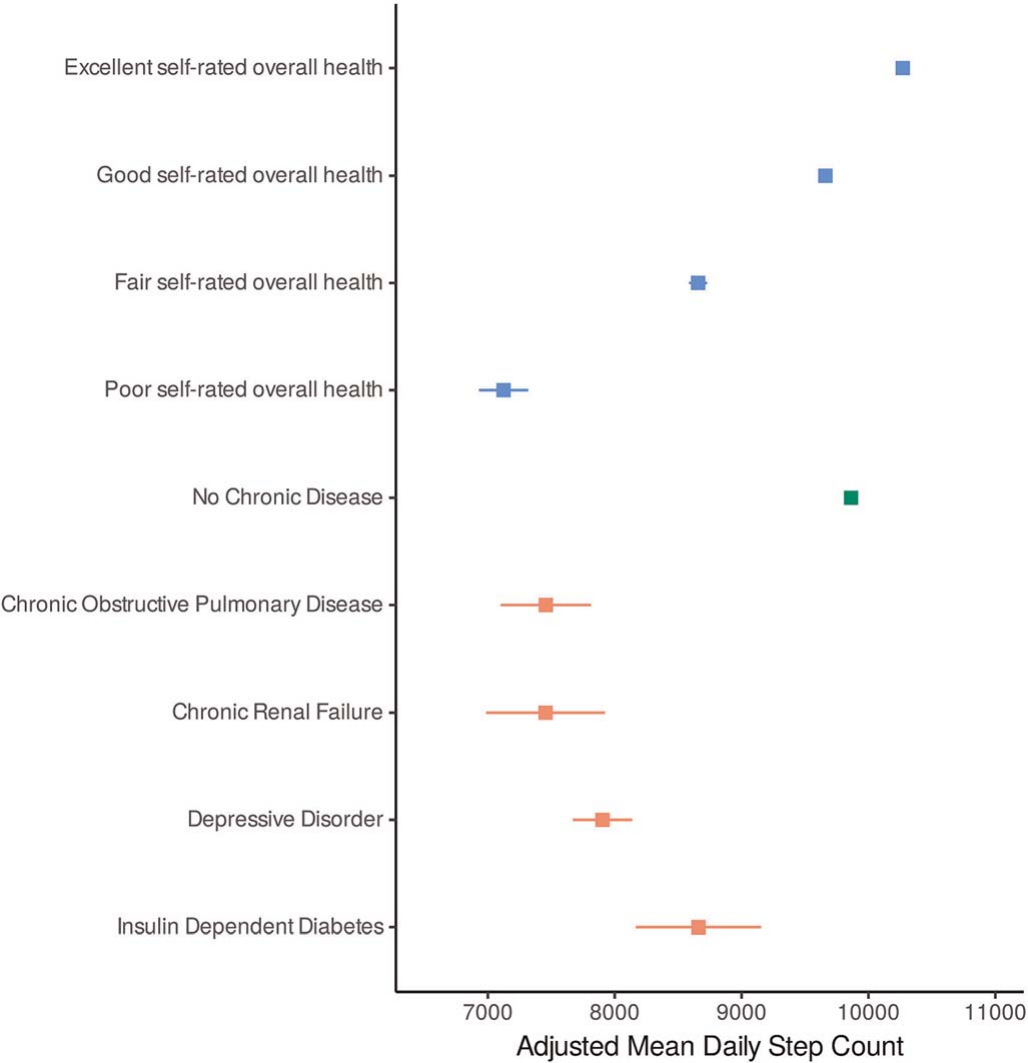
Adjusted estimated marginal mean (95% CI) daily step count according to self-reported overall health status, hospital data–derived chronic disease status, and select diagnoses for 75,263 UK Biobank participants. Mean daily step counts are adjusted for age and sex.

Baseline data from 75,263 UK Biobank participants without prevalent CVD or cancer are presented in Table 1 and Supplemental Figure 2 (Flow diagram for descriptive and survival analyses in UK Biobank physical activity cohort, http://links.lww.com/MSS/D29). Peak step cadence demonstrated expected variations by self-reported usual walking pace (Supplemental Fig. 3, Peak step cadence in the UK Biobank Physical Activity Cohort, summarized by self-reported walking pace and sex, http://links.lww.com/MSS/D29) and our measurements of steps demonstrated orthogonality to standard overall acceleration and time-use metrics (Supplemental Fig. 4, Spearman’s rank correlation plot between accelerometer-based physical activity metrics, http://links.lww.com/MSS/D29). Participants who self-reported that their overall health was excellent were more active than all other participants, taking 3091 more steps (95% confidence interval (CI), 2826–3356; *P* < 0.001) than those reporting that their overall health was poor. Similarly, self-reported brisk walkers had a peak 1-min cadence 13.9 steps per minute (95% CI, 13.3–14.4; *P* < 0.001) higher than slow walkers. Adjusted mean daily step counts by self-reported health status and by selected physician-diagnosed chronic conditions are presented in Figure 3.

**TABLE 1:** Overall physical activity metrics by demographic characteristic in the UK Biobank.

Characteristic	*N* (%)	Daily Steps	Peak Cadence (Steps per Minute)	Overall Acceleration (m*g*)
Overall	75,263 (100.0)	9156 (6936–11,762)	113 (106–120)	27.5 (22.9–32.9)
Sex				
Female	43,670 (58.0)	9060 (6878–11,600)	115 (107–122)	27.8 (23.3–33.2)
Male	31,593 (42.0)	9278 (7022–12,000)	111 (104–117)	27.0 (22.3–32.6)
Age, yr				
40–49	7204 (9.6)	9406 (7144–12,070)	116 (109–123)	30.4 (25.5–36.5)
50–59	23,293 (30.9)	9250 (7058–11,937)	115 (108–122)	29.1 (24.4–34.8)
60–69	32,821 (43.6)	9246 (7022–11,828)	112 (105–119)	26.9 (22.5–32.1)
70–79	11,945 (15.9)	8578 (6416–10,992)	109 (102–116)	24.6 (20.5–29.2)
Ethnicity				
Non-White	2370 (3.1)	8796 (6567–11,446)	114 (106–121)	28.7 (23.9–34.2)
White	72,893 (96.9)	9166 (6948–11,773)	113 (106–120)	27.5 (22.9–32.9)
Body mass index				
Underweight (<18.5 kg·m^−2^)	443 (0.6)	10,036 (7812–12,860)	119 (110–125)	31.3 (25.2–36.8)
Normal weight (18.5–24.9 kg·m^−2^)	30,214 (40.1)	9790 (7580–12,402)	116 (109–123)	29.5 (24.7–35.1)
Overweight (25.0–29.9 kg·m^−2^)	30,705 (40.8)	9148 (6990–11,695)	112 (106–119)	27.0 (22.7–32.1)
Obese (30+ kg·m^−2^)	13,901 (18.5)	7696 (5697–10,140)	109 (101–115)	24.4 (20.3–29.2)
Education				
School leaver	16,670 (22.1)	8729 (6562–11,350)	112 (104–119)	27.0 (22.3–32.5)
Further education	24,960 (33.2)	8990 (6755–11,615)	112 (105–119)	27.5 (22.9–32.9)
Higher education	33,633 (44.7)	9482 (7262–12,034)	114 (107–121)	27.7 (23.2–33.1)
Smoking status				
Never	44,097 (58.6)	9240 (7048–11,816)	114 (107–121)	27.8 (23.2–33.2)
Former	26,035 (34.6)	9108 (6884–11,767)	112 (105–119)	27.3 (22.7–32.7)
Current	5131 (6.8)	8584 (6308–11,240)	110 (103–117)	26.3 (21.5–31.8)
Alcohol consumption				
Never	34,216 (45.5)	8848 (6656–11,431)	113 (106–120)	27.3 (22.6–32.7)
<3 d·wk^−1^	36,977 (49.1)	9497 (7282–12,077)	113 (106–120)	27.8 (23.3–33.2)
3+ d·wk^−1^	4070 (5.4)	8622 (6266–11,420)	112 (104–120)	26.8 (21.8–32.4)
Townsend deprivation				
Least deprived (<−3.8)	18,806 (25.0)	9149 (7040–11,734)	112 (105–119)	27.6 (23.1–32.9)
Second least deprived (−3.8 to −2.2)	18,810 (25.0)	9140 (6958–11,645)	112 (106–119)	27.5 (23.0–32.9)
Second most deprived (−2.5 to −1.2)	18,831 (25.0)	9162 (6911–11,752)	113 (106–120)	27.5 (22.9–32.9)
Most deprived (≥−0.2)	18,816 (25.0)	9170 (6828–11,918)	114 (107–121)	27.4 (22.7–32.9)
Self-reported usual walking pace				
Brisk	36,626 (48.7)	9680 (7466–12,288)	115 (108–122)	29.1 (24.4–34.6)
Steady	35,614 (47.3)	8806 (6661–11,354)	111 (104–118)	26.4 (22.0–31.4)
Slow	2896 (3.8)	6608 (4434–9136)	103 (94–112)	22.3 (18.1–27.2)
None of the above	60 (0.1)	5602 (3320–9884)	101 (92–108)	22.6 (18.9–28.5)
Missing	67 (0.1)	3280 (959–5460)	87 (60–96)	18.9 (14.1–24.2)
Self-reported overall health				
Excellent	17,730 (23.6)	9786 (7597–12,389)	115 (108–123)	29.2 (24.5–35.0)
Good	45,611 (60.6)	9178 (6998–11,772)	113 (106–120)	27.5 (23.0–32.7)
Fair	10,484 (13.9)	8222 (6016–10,776)	110 (102–117)	25.3 (20.9–30.4)
Poor	1438 (1.9)	6616 (4377–9268)	105 (95–112)	22.9 (18.4–28.1)
Wear season				
Spring	17,269 (22.9)	9293 (7060–11,932)	113 (106–120)	27.9 (23.2–33.3)
Summer	19,947 (26.5)	9601 (7312–12,267)	112 (105–119)	28.1 (23.4–33.6)
Autumn	22,286 (29.6)	9056 (6878–11,582)	113 (106–120)	27.4 (22.9–32.8)
Winter	15,761 (20.9)	8600 (6509–11,130)	114 (106–121)	26.6 (22.1–31.7)

Activity metrics reported as unadjusted median (interquartile range).

### Association of step counts with all-cause and cardiovascular mortality

**FIGURE 4:**
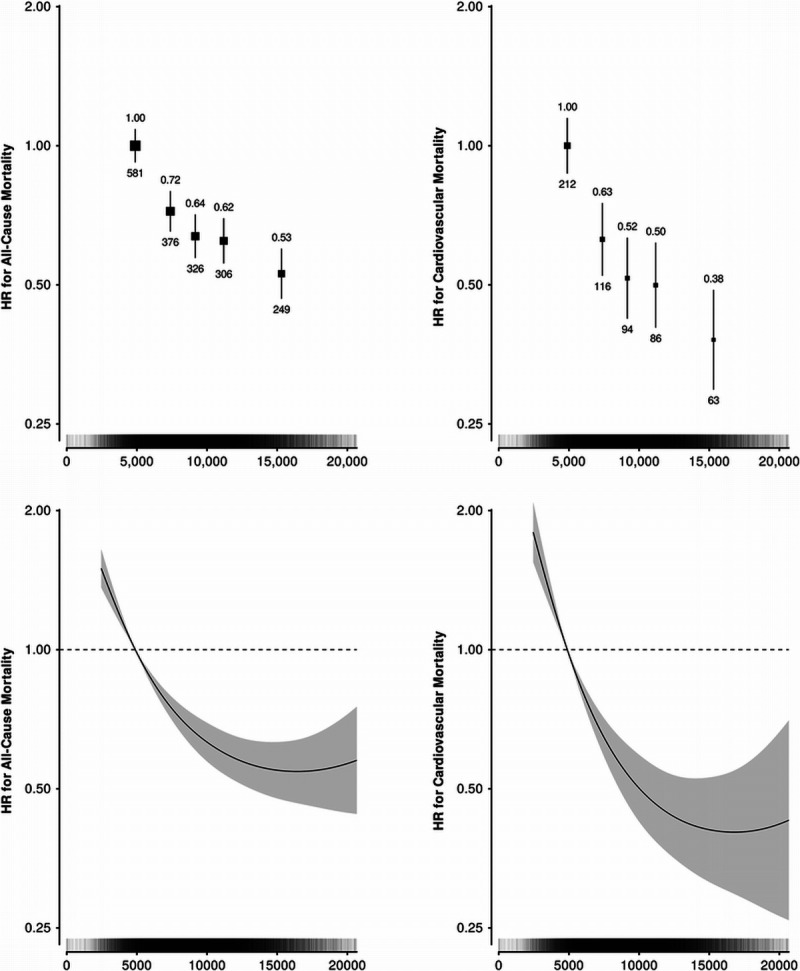
Top: Forest plots for all-cause mortality and CVD mortality associations with quintiles of daily step count. Bottom: continuous daily step count for 75,263 UK Biobank participants. Hazard ratios (HR) and 95% CI were calculated using age as a timescale, adjusted for sex, ethnicity, education, alcohol intake, smoking status, Townsend deprivation index, processed meat intake, fresh fruit intake, oily fish intake, and added salt intake. HR is above and number of events is plotted below each data point. Spline plot of hazard ratio and 95% CI of the association of continuously modeled median daily step count. Vertical bars along the step axis indicate distribution of participant daily step counts.

The 75,493 participants had a median follow-up of 6.9 (interquartile range, 6.3–7.4) yr, with 572 events in the CVD mortality analysis and 1844 events in the all-cause mortality analysis (Fig. 4). For CVD mortality, an association between higher daily step count and lower hazard ration was observed. A median daily step count of 8278 to 10,081 steps per day was associated with a 48% (37%–58%) lower risk of CVD mortality compared with participants taking fewer than 6430 steps per day, whereas taking 12,482 or more steps was also associated with a 62% (51%–70%) lower risk on CVD mortality. Similar results were observed in the analysis of all-cause mortality and median daily step count, with a 36% (29%–43%) and 47% (40%–53%) lower risk of all-cause mortality in the middle and most active 20%, respectively.

## DISCUSSION

We have developed a new open-source step counting method, informed by self-supervised machine learning methods that substantially outperforms current wrist-worn step counting algorithms in the free-living environment. The open data and code released with this manuscript will provide the global research community access to a more transparent and well-validated method to measure steps in large-scale wrist-worn accelerometer datasets. When applying the algorithm and resulting step metric in epidemiological analysis, we demonstrated that a higher daily step count is associated with a lower risk of all-cause and cardiovascular mortality.

Our novel approach of using a hybrid step detection model that involves self-supervised machine learning outperformed existing wrist-worn step counting methods, producing a 12.5% MAPE and 1.3% step underestimation during free living. Wrist-worn step counting is highly popular in both commercial and research applications, but valid step detection at the wrist can be associated with high measurement error relative to ground truth. In 2018, Toth et al. (6) assessed wrist-worn step detection in free-living conditions, finding error rates between 18% and 120% across a range of methodologies. We found similar performance in current open-source algorithms during free-living testing, with a mean average percent error ranging from 64% to 231%. Even while analyzing data from a different device, sampling rate, and wrist location than the training data, external validation of the hybrid self-supervised model in the Clemson dataset demonstrated a 9.2% error during regular walking in the laboratory-based setting, below the 10% MAPE threshold required during treadmill-based validation (11). External validation of the hybrid self-supervised model outperformed both reference algorithms, including the Verisense algorithm, which was trained and tuned using the Clemson laboratory dataset (10,25).

This study demonstrates a strong inverse curvilinear association between increased step count and lower risk of fatal CVD and all-cause mortality while highlighting the importance of accurate step detection algorithms in epidemiological analysis. Our current results parallel those of Paluch et al. (15), who demonstrated that higher daily step counts are associated with an incrementally lower risk of all-cause mortality across 15 international longitudinal cohorts nearly exclusively using hip-mounted devices. Using less accurate step-detection methods, another study has also indicated a curvilinear association between daily steps and CVD mortality (14). Although the direction of epidemiological associations may remain broadly similar across step detection algorithms, it is important that algorithms derive step counts as accurately as possible. Accurate step counting will be particularly important when translating results into target levels of physical activity in guidelines compatible with device-measured activity (31). Reporting of inaccurate step counts may additionally be demotivating and counterproductive in terms of health metrics and behavioral change for individuals monitoring their own physical activity (32). It is important to note, however, that these guidelines will be translated into everyday use by the public using commercial activity trackers. Although the authors believe that guidelines should be based on the most accurate step counts available in current epidemiological research, commercial step detection methods will inherently differ from those used in the current study.

Clear strengths of our study include the development of a high accuracy step counting algorithm trained on a free-living dataset of step-annotated wrist-worn accelerometer data. Although these training data consisted of short 1-h data collection windows, it is important to note that the OxWalk dataset is the largest open-source, free-living dataset currently available with video annotations of ground-truth steps. This enables algorithm assessment against gold-standard ground truth in place of using a secondary device as the reference criterion (33,34). Some overestimation of step counts may occur when applied to multiday protocols due to the lack of extended periods of sedentary inactivity in the short training data, however; class rebalancing was utilized to minimize this effect. The OxWalk dataset and UK Biobank datasets are not representative samples of the UK population in terms of socioeconomic and ethnic backgrounds, which may insert bias into subsequent use of the step and gait classification in other populations.

## CONCLUSIONS

We have developed a new, open, and transparent method that markedly improves the ability to measure steps in large-scale wrist-worn accelerometer datasets. This validated algorithm can be used widely for other wearable studies, and an open-source, reproducible pipeline is provided to facilitate implementation. While using this validated step detection method trained using free-living data, we demonstrate an inverse dose–response of daily step count with all-cause and CVD mortality as a test of algorithm face validity. This reinforces public health messaging for increasing physical activity and lays additional groundwork toward the incorporation of a target daily step count into public health guidelines in a population increasingly open and interested in using wearables for personal activity tracking.
